# Supplementary material for: Causal interplay between lactose intolerance and gut microbiota: a combined bidirectional Mendelian randomization and in vivo validation study
Source: Front Nutr. 2026 Jun 1;13:1803337. doi: 10.3389/fnut.2026.1803337 (PMC13265576; doi:10.3389/fnut.2026.1803337)
Supplement: Supplementary file 1 [file Data_Sheet_1.zip › supplementary materials/Forward/leave one out cross validation/ebi-a-GCST90027486.finngen_R12_E4_LACTONAS.pdf]

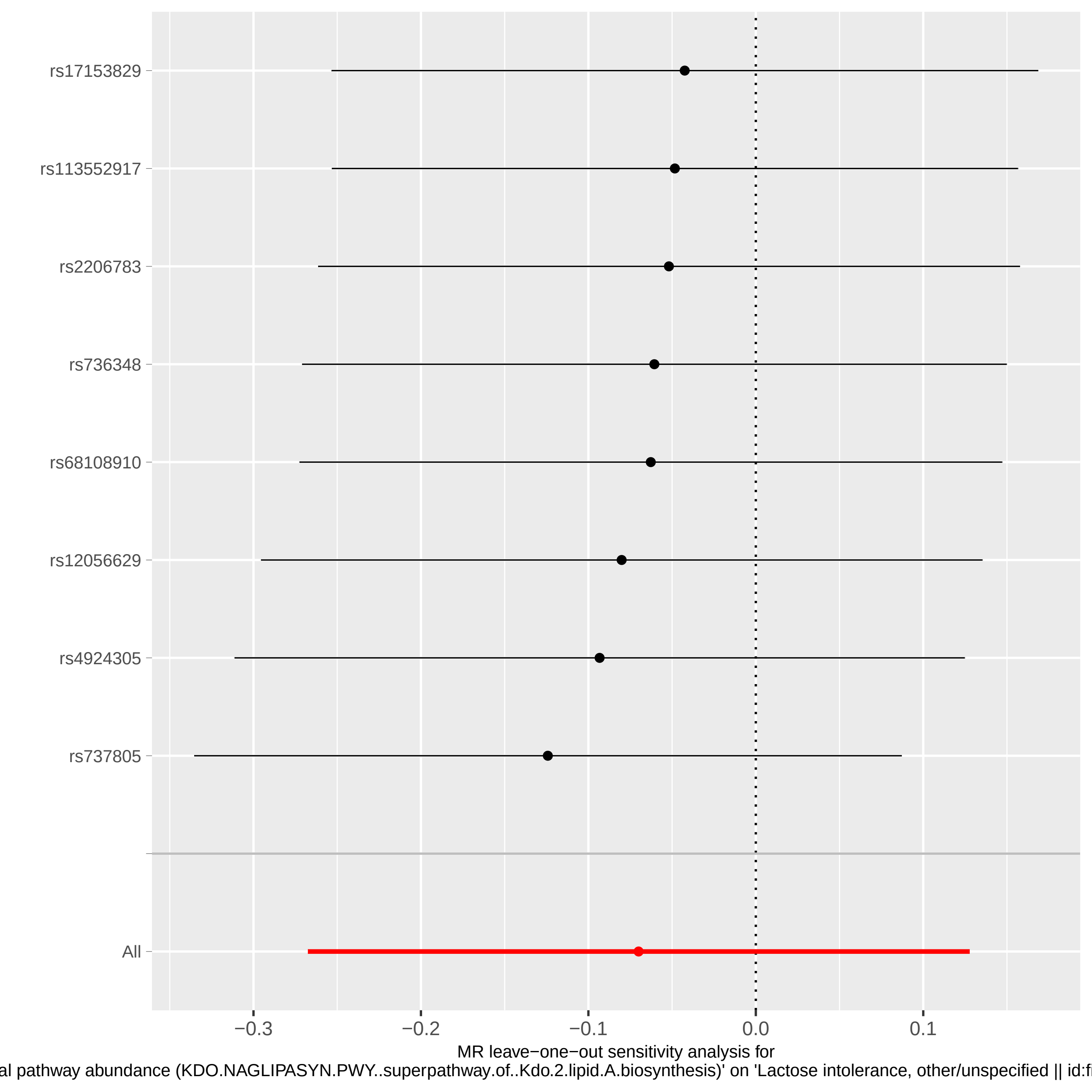

rs17153829
rs113552917
rs2206783
rs736348
rs68108910
rs12056629
rs4924305
rs737805
All
−0.3
−0.2
−0.1
0.0
0.1
MR leave−one−out sensitivity analysis for
al pathway abundance (KDO.NAGLIPASYN.PWY..superpathway.of..Kdo.2.lipid.A.biosynthesis)' on 'Lactose intolerance, other/unspecified || id:f
